# The Task-Based fMRI Using von Zerssen Scale in Recurrent Depression Disorder: A Replication Study

**DOI:** 10.1155/da/2617054

**Published:** 2025-05-01

**Authors:** Alexander Korotkov, Artem Myznikov, Anastasia Komarova, Elena Isaeva, Irina Solnyshkina, Denis Cherednichenko, Michael Didur, Maxim Kireev

**Affiliations:** ^1^Interdisciplinary Brain Research Department, N.P. Bechtereva Institute of Human Brain Russian Academy of Science, Saint Petersburg, Russia; ^2^Department of General and Clinical Psychology, The Pavlov First Saint Petersburg State Medical University, Saint Petersburg, Russia; ^3^Institute of Cognitive Studies, Saint Petersburg State University, Saint Petersburg, Russia

**Keywords:** depression, fMRI, local neural activity, self-rating scale, translational cross-validation paradigm

## Abstract

Currently, translation into clinical practice of scientific knowledge on pathological reorganization of brain mechanisms in psychiatric disorders, as suggested by functional neuroimaging data, remains limited. This situation calls for the exploration of new approaches, which were recently proposed for the combined use of the simultaneous application of psychodiagnostic testing and fMRI scanning. Consequently, a self-rated psychodiagnostic scale was used as an experimental task during fMRI scanning for patients with major depressive disorder. Given the promising neuroimaging results obtained in these studies, in current research, our objective was to replicate these results and conduct an fMRI study using statements from the von Zerssen depression as experimental conditions. Eighteen patients with recurrent depressive disorder and healthy volunteers participated in the study to replicate the group size of previous research. The results obtained showed that patients with recurrent depressive disorder exhibited greater activity in the right precuneus and bilateral supramarginal gyrus than healthy controls while responding to diagnostically specific (DS) statements compared to diagnostically neutral (DN) ones. These findings replicate the main results of the original study and emphasize the potential of this approach in the field of translational psychiatry. In addition, they contribute to understanding the pathophysiological mechanisms of depression through the use of this unique fMRI paradigm.

## 1. Introduction

Functional neuroimaging is a powerful tool for studying brain mechanisms in healthy and diseased states and is widely used in diagnostics in numerous clinical disciplines but not in psychiatry [1]. In accordance with modern concepts, any human activity and behavior are maintained by dynamically organized and spatially distributed functional neuroanatomical systems that are characterized by their anatomical composition, the level of functional activity of their elements, and connections between them. The modern literature also contains a large amount of neuroimaging data that describes both the activity of such systems in a healthy brain and their reorganization in psychiatric diseases [2–4]. Based on accumulated data, numerous models of the reorganization of brain work during the development of mental disorders have been created. From the literature, one can see the discrepancy between deep scientific ideas about pathological reorganization of the brain mechanisms and the practical application of this knowledge. Attempts to translate this knowledge into clinical practice are still not effective enough, and currently, no diagnostic criteria have been developed to use them in routine clinical work in psychiatry. At the same time, recently, the translational cross-validation paradigm was proposed, which assumes the use of psychodiagnostic tests, namely self-rating scales, as tasks during fMRI were proposed [5]. In addition to the cross-validation of psychodiagnostic instruments and functional imaging data, this approach should help solve some significant problems in this field. For example, problem related to the validation of different methods within fMRI modality (activation studies with tasks, task-modulated functional connectivity, and resting state functional connectivity).

The efficiency of this approach was proven by the authors in two recent publications. In the first fMRI study [5], in a sample of patients with depressive episodes within the framework of major depressive disorder and bipolar affective disorder, statements from the von Zerssen questionnaire were used as tasks during fMRI. In a group of patients compared to the healthy control group, an increase in neuronal activity of various brain structures (pre- and postcentral right gyrus, left inferior and middle frontal gyrus, and left middle temporal gyrus) was observed during the processing of diagnostically specific (DS) statements compared to those that were diagnostically neutral (DN). In another study [6], the same approach was used to study patients with schizophrenia and major depressive disorder. Statements specific to depression and related to paranoia were used as experimental conditions during fMRI. In patients with schizophrenia, the spatial distribution of the brain regions, including the right angular gyrus, the left posterior cingulate gyrus, and the precuneus, whose activity increased when responding to statements specific to depression and paranoia, differed from that of a group of patients with depression compared to a healthy control group. As in the first study, significant differences were obtained for the patient groups compared to healthy controls. However, comparison among the patients themselves did not reveal statistically significant differences.

These results inspired us to replicate the study by Stoyanov et al. [5] on independent samples of patients with depressive disorder and healthy subjects. In the current study, we used diagnostically significant statements from the von Zerssen questionnaire translated into Russian and DN statements translated from [5] as tasks for the fMRI scanning of patients with recurrent depressive disorder and healthy subjects. Given the specifics of the task, we expected to see a difference in the activity of structures involved in self-referencing processes during the evaluation of DS statements that could reflect the degree to which they correspond to the internal representations of patients with recurrent depression and healthy controls.

## 2. Materials and Methods

### 2.1. Participants

Twenty patients (M:F = 12:8, age 31.7 ± 4.1 years) with verified recurrent depressive disorder (F33) were included in this study. The diagnosis of depression was established by a qualified and licensed psychiatrist at the District Psycho-Neurological Outpatient Department. Patients were excluded if they had psychotic, anxiety, or substance-related disorders, a severe somatic disorder with decompensation, a neurological disorder, or a history of head trauma resulting in loss of consciousness. All patients, for the moment of the fMRI scan, took selective serotonin reuptake inhibitors (SSRIs). Twenty healthy right-handed volunteers (M:F = 8:12, age 23.3 ± 4.1 years) without a history of psychiatric or neurological disease or current medication intake were recruited through an advertisement placed on social media and participated in the experiment for a monetary reward (1500 rubles). We evaluated the handedness of the participants using the Edinburgh Handedness Inventory [7]. All participants provided their informed consent in writing prior to starting the study. We performed all procedures in accordance with the Declaration of Helsinki, and they were approved by the Ethics Committee of the N.P. Bechtereva Institute of the Human Brain, St. Petersburg, Russia.

### 2.2. Stimuli and Procedure

In the current study, we used statements from the von Zerssen Depression Scale to reproduce the findings of the article that we are replicating [5]. This scale consists of 16 statements, and patients indicate their level of agreement using the following options: “completely true,″ “mostly true,″ “partly true,″ or “not true.” The scoring system for each item on the von Zerssen Depression Scale is detailed in Table 1. The statements of the Depression Scale were labeled DS. For the control condition, we used 16 statements that were considered DN and were associated with general interests and likes (“I like to assemble furniture”) (Table 1). Before the scanning procedure, each participant was instructed to read the statements carefully and select the response that best reflected their level of agreement with each statement using the manipulator buttons. During fixation periods, participants were required to fix their gaze on a cross displayed on the screen.

A block design was used for the fMRI task, consisting of blocks that included statements from DS and DN interspersed with visual fixation blocks (OFF). The order of the blocks within the session was consistent (DS–OFF–DN–OFF, etc.), but the statements within the blocks were presented in random order. Each block contained four statements, each displayed for 8 s, resulting in 32 s for both DN and DS blocks. There was a 4 s delay between the start of the scan and the first block. Each statement was centered on the screen, while the answer choices were positioned in the four corners. To counterbalance the placement of the responses on the screen, two session options were created:

1. Left–right (LR): completely true = upper left; mostly true = lower left; somewhat true = lower right; not true = upper right.
2. Right–left (RL): completely true = upper right; mostly true = lower right; somewhat true = lower left; not true = upper left.

**Figure 1:**
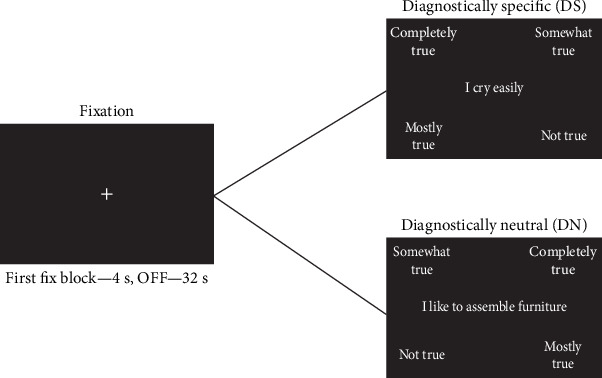
Schematic representation of fMRI task using both diagnostically specific (DS) and diagnostically neutral (DN) statements. Left–right (LR) variants of response placement are presented for the DS probe and right–left (RL)—for the DN probe. OFF, fixation block.

The schematic representation of the task is presented in Figure 1. We calculated the reaction times for both the DS and DN statements and the sum of scores for the von Zerssen scale. We excluded two patients from the analysis due to low DS scores (DS = 5 and DS = 6) as well as two healthy controls due to high DS scores (DS = 22 and DS = 15); a total of 18 healthy controls and 18 patients were included in the analysis.

### 2.3. fMRI Image Acquisition Procedure and Image Processing

fMRI data were recorded using a 3 T Philips Achieva scanner. Structural images were acquired before the task using a T1-weighted pulse sequence (T1W-3D-FFE; repetition time [TR] = 25 ms; echo time [TE] = 2.2 ms; 30° flip angle), measuring 130 axial slices (field of view [FOV] = 240 × 240 mm) of 1 mm thickness and a 1 × 1 mm pixel size. Functional images were obtained using an echo-planar imaging sequence (TR = 2 s, TE = 35 ms; 90° flip angle; FOV = 200 × 186 mm). In total, 32 continuous 3.5 mm thick axial slices (voxel size = 3 × 3 × 3.5 mm) covering the entire cerebrum and most of the cerebellum were oriented with respect to structural images. To achieve steady-state magnetization, the four “dummy” scans were performed at the beginning of the fMRI session.

An MR-compatible cervical collar was used to prevent head movements. Data preprocessing and subsequent statistical analyses were performed using SPM12 software (Statistical Parametric Mapping, http://www.fil.ion.ucl.ac.uk/spm/) run in MATLAB R2017a (Mathworks Inc., Natick, MA, United States). The preprocessing of raw fMRI data for each participant included the following stages: realignment, slice-time correction, coregistration, segmentation, normalization, and smoothing (8 mm FWHM). During the realignment stage, six parameters of head movement relative to the first image (translations and rotations on three coordinate axes) were generated. For head motion control, we calculated framewise displacement (FD) based on the method suggested by Jenkinson et al. [8]. An FD > 0.5 mm was used as an exclusion criterion for both the patient and healthy control groups. According to this criterion, all participants were included in the study. The mean FD for the patient group was 0.16 ± 0.09, while the mean FD for the healthy control group was 0.12 ± 0.04.

### 2.4. Statistical Analysis

On the first level, individual general linear models (GLMs) were generated for each participant and patient. The GLM included regressors for both DS and DN blocks, as well as six parameters of head movement. The regressors were then convolved with the standard hemodynamic response function (HRF). The default 128-s high pass filter was applied for removing low-frequency noise. Second, the beta values of the regression coefficients for the regressors in GLMs were estimated at the individual level of analysis. Linear *t*-contrasts “DS > DN” for each subject were calculated and used as a variable for the second-level analysis. On the second level, we used a two-sample *t*-test with DS > DN contrasts for comparison groups of healthy participants and patients. Statistical parametric maps were created with the uncorrected *p*-value < 0.001 and a subsequent cluster-level family-wise error (FWE) correction with *p* < 0.05. The SPM results were visualized using the MRIcron toolbox (https://www.nitrc.org/projects/mricrogl). The REX toolbox (http://www.nitrc.org/projects/rex) was applied to illustrate differences in values of beta coefficients in obtained clusters of changes in the BOLD signal.

## 3. Results

### 3.1. Behavioral and Psychodiagnostic Results

Demographic, behavioral, and psychodiagnostic data for the compared groups are presented in Table 2. There were significant differences in age, reaction time for DS probes, and von Zerssen score between the groups. The groups did not differ in the gender distribution and reaction time for the DN probes. Since the groups differed significantly in age, we performed the analysis of fMRI data both with and without age as a covariate of noninterest.

### 3.2. fMRI Results

**Figure 2:**
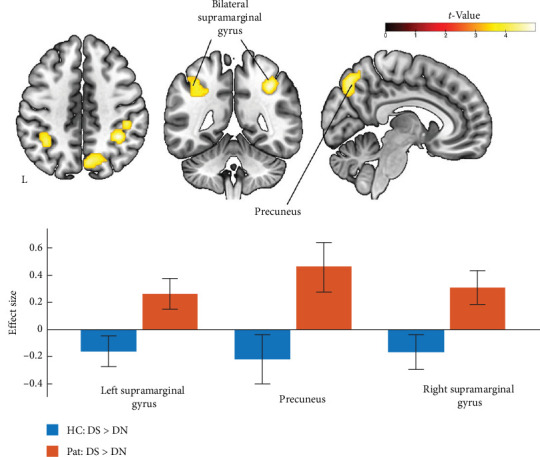
Results of fMRI data analysis: Pat > HC (2-sample *t*-test) using individual DS > DN contrasts, voxel-level *p* < 0.001 uncorrected, cluster-level pFWE < 0.05. Histograms of effect size were created based on the flexible factorial model. DN, diagnostically neutral; DS, diagnostically specific; HC, healthy control; Pat, patients.

**Figure 3:**
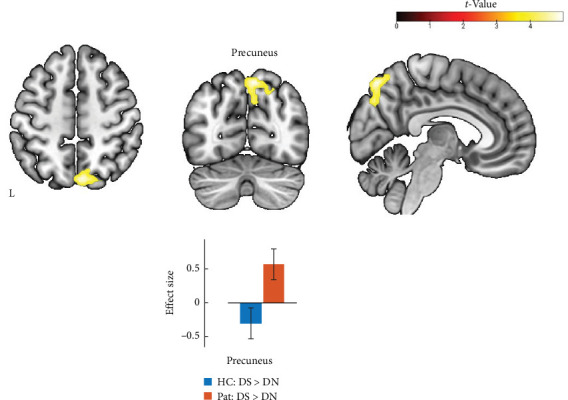
Results of fMRI data analysis: Pat > HC (2-sample *t*-test) using individual DS > DN contrasts with age as covariate, voxel-level *p* < 0.001 uncorrected, cluster-level pFWE < 0.05. Histograms of effect size were created based on the flexible factorial model. DN, diagnostically neutral; DS, diagnostically specific; HC, healthy control; Pat, patients.

Two-sample *t*-tests for comparison between the healthy participants' group and MDD patients in DS > DN contrast revealed increased activity in the bilateral supramarginal gyrus and precuneus in the group of patients with recurrence of the depressive disorder compared to the healthy control group (Figure 2 and Table 3). These results align with a previous study [5], in which an increase in brain activity was anticipated and observed in the patient's group. Moreover, the cluster on the right IPL/SPL had a location similar to those described in the work mentioned above. The histograms for the effect size for both conditions and both groups are illustrated in Figure 2. After using age as a covariate in the same statistical second-level model, only the cluster in the right precuneus survived after correction for multiple comparisons (Figure 3 and Table 3).

## 4. Discussion

The translational cross-validation approach that involves the use of psychodiagnostic questionnaires during a task-based fMRI study [5] demonstrated a promising direction for research to bridge the gap between psychiatry and neuroimaging. In light of this, we performed a replication fMRI study using the same stimulus material in independent samples from patients with recurrent depressive disorder and healthy controls. Our findings revealed that activity in certain brain regions, such as the right precuneus and the bilateral supramarginal gyrus, was significantly higher in depressed patients compared to healthy subjects when responding to DS statements versus DN ones. When we used the age as a covariate of noninterest, only the result in the right precuneus survived after correction for multiple comparisons. These results align with those of the previous study, which also indicated (1) increased activity in multiple brain structures among the patient group and (2) overlapping results in the area of the right supramarginal gyrus in both the previous and replication studies. This consistency emphasizes the effectiveness of such a psychodiagnostically based fMRI method as the study approach in distinguishing between the patient group and healthy controls.

The current study identified an increase in local activity in the bilateral supramarginal gyrus and the right precuneus. There is ample evidence in the literature that depression is associated with structural and functional changes in the lateral and medial parietal cortex [9–12]. These regions of the brain regions are known to be associated with working memory. Working memory generally refers to a set of processes that temporarily store and manipulate information to maintain goal-directed behavior [13]. Research shows that people with depression may have reduced working memory capacity [14, 15]. The findings of the current study can reflect some adaptive mechanisms in patients with depression to overcome inefficiencies in working memory processing. In a study that used a working memory task, overactivity in brain structures involved in working memory, including the dorsolateral prefrontal cortex and anterior cingulate cortex, was shown in patients with major depressive disorder [16]. In addition, there is evidence for the involvement of the precuneus in memory-related imagery processes [17]. Imagery and working memory play a key role in the retrieval of autobiographical information [18], which is closely related to the task used in this study. However, it should be noted that working memory deficit is not unique to depression and can be associated with severe psychopathology in general (for example, schizophrenia or anxiety disorders) [19].

Working memory is crucial for effective emotion regulation, mainly through selective attention and distraction [20]. Selective attention involves directing the focus toward or away from specific stimuli or their attributes, while distraction refers to the limitation of attention to an external stimulus by concentrating internally on information held in working memory [21, 22]. Working memory capacity is also crucial for regulating emotions and overcoming cognitive biases since it allows people to focus on relevant emotional information that supports their goals while ignoring distractions [23, 24]. Research indicates that people with depression and those in remission are more likely to use maladaptive emotion regulation strategies, such as rumination, and less likely to use adaptive strategies, such as cognitive reappraisal [25]. Rumination involves repetitive thinking or dwelling on negative feelings and distress and their causes and consequences [26]. This maladaptive emotion regulation strategy is potentially associated with disturbances within brain regions of the default mode network, including precuneus and bilateral supramarginal gyrus [27].

However, the functional specialization of the precuneus and supramarginal gyrus should be considered in conjunction with the activity in which the subjects were engaged, namely by attributing the statements presented on the screen to themselves and by expressing their agreement with them. In such a view, the structures mentioned above, being the nodes of the default mode network, are more likely to be involved in processes related to self-referencing [28]. Depressed patients are more likely to attribute DS statements of the von Zerssen questionnaire to themselves, which can be associated with a higher level of activity in patients compared to controls. Increased activity or less deactivation of structures attributed to the DMN network has been widely described in MDD, for example, in the evaluation of emotional stimuli [29–31], rumination [27], and self-referencing-related processes [32]. Marchetti et al. [33] tried to implement these findings in a general framework, suggesting the overpowering of task-positive (TP) activity by task-negative (TN) activity as a potential mechanism of DMN involvement in the pathogenesis of depression. From this point of view, the various features of depression, such as rumination, increased self-focus, and poor attentional control, can be explained by the aberrant interactions between TP and TN brain systems. However, since we did not perform a task-modulated functional connectivity analysis between revealed clusters and other brain regions, we cannot speculate about current findings as the appearance of a TP-TN imbalance.

We did not observe any activations in the motor cortex, as previously described in the original study. We believe that the main reason for this is the difference in the study design, namely the counterbalancing of answer placement on the screen we used. In the original research, the fixed placement of the answers on the screen (completely true = upper left; mostly true = lower left; somewhat true = lower right; not true = upper right) was used. In such stimuli appearance, healthy controls responded to DS statements, more likely by choosing an answer located on the right side of the screen (either “not true” or “somewhat true”), which required them to press buttons with their right hand. In contrast, patients with depression tended to select positive answers on the left side of the screen, requiring them to use their left hand. A comparison of groups of patients and healthy controls using the DS > DN contrast would likely result in residual activation in the left motor cortex, as observed in the original article. However, in our current study, we used two variations of answer placement on the left and right sides of the screen that mitigated this effect.

## 5. Limitations

The current study has some limitations. First, the age difference between the patient group and the healthy control group was significant. When we applied age as a covariate of noninterest in the statistical analysis, the clusters in the supramarginal gyrus did not survive the correction for multiple comparisons: only the result in the right precuneus was statistically significant. This fact may be due to (1) age-related changes in activity and (2) a decrease in degrees of freedom. Second, the data about patient medications were not analyzed. This can be critical since taking SSRIs in patients with depression can modulate the local activity of brain structures [34, 35] and functional connectivity [36–38]. On the one hand, the SSRI treatment led to an increase in local neural activity in DMN nodes (anterior/posterior cingulate cortex) [39] and an increase in functional connectivity between the anterior cingulate cortex and the limbic regions during the processing of positive emotional stimuli [40]. However, the meta-analysis of Delaveau et al. [41] demonstrated that DMN-associated regions during SSRI treatment demonstrated decreased activation during emotional activation studies. Since we obtained a higher level of local neuronal activity of the precuneus in patients within the current study, this fact does not exclude the influence of SSRI therapy. Future investigations based on the described approach should include information about SSRI doses that can be incorporated as additional covariates in the statistical analysis of fMRI data. In addition, increasing the sample size of both the patients and healthy control groups is expected to increase the validity of the results. Finally, applying new statistical methods for patient—healthy control comparison, such as normative modeling [42], significantly increased the opportunity for the application of the current fMRI results in clinical practice.

## 6. Conclusions

Thus, the results obtained in the current replication study allow us to confirm the main findings of the study we replicated [5] and indicate the effectiveness of an approach that involves the application of psychodiagnostic tools as stimulus material in fMRI studies. Using the approach, characterized by the registration of local neural activity by fMRI during the completion of psychodiagnostic tests, allows us to obtain unique integral information about the primary disease and potentially improve the diagnostic framework in psychiatry of depression.

## Figures and Tables

**Table 1:** The list of diagnostically specific (DS) and diagnostically neutral (DN) statements and scores for DS statements.

Diagnostically neutral statements	Diagnostically specific statements	Scores for DS statements			
		Completely true	Mostly true	Partly true	Not true
1. I like assembling furniture	1. I enjoy all sorts of games and pastimes	0	1	2	3
2. I like laying bricks or tiles	2. I am more sensitive to criticism than I used to be	3	2	1	0
3. I like developing new medicines	3. Lately, I have been very anxious and easily startled	3	2	1	0
4. I like studying ways to reduce water pollution	4. I cry easily	3	2	1	0
5. I like writing books or plays	5. I am afraid of losing my mind	3	2	1	0
6. I like playing a musical instrument	6. I feel down and depressed	3	2	1	0
7. I like giving individual exercises to everyone	7. I cannot understand what I read as well as I used to	3	2	1	0
8. I like helping people with personal or emotional problems	8. I would like most of all to take my own life	3	2	1	0
9. I like buying and selling stocks and bonds	9. In the morning, I feel particularly bad	3	2	1	0
10. I like running a shop	10. I no longer have any relationship with others	3	2	1	0
11. I like creating spreadsheets using computer software	11. I feel that I am about to go to pieces	3	2	1	0
12. I like keeping records or filling out various forms	12. I am constantly afraid of saying or doing something wrong	3	2	1	0
13. I like repairing household appliances	13. I am much less interested in my love life now than I used to be	3	2	1	0
14. I like raising fish at a fish farm	14. I often feel simply miserable	3	2	1	0
15. I like conducting chemistry experiments	15. As hard as I try, I cannot think logically at all	3	2	1	0
16. I like studying the movement of the planets	16. I no longer have any feelings	3	2	1	0

**Table 2:** Demographic, behavioral, and psychodiagnostic data for groups of healthy control and patients.

Characteristic	Healthy	Patients	*p*-Value
Age	23.3 ± 4.1	31.7 ± 4.1	0.001
Reaction time (ms), DS	3265 ± 511	3910 ± 681	0.002
Reaction time (ms), DN	3428 ± 662	3506 ± 638	0.72
Von Zerssen Score	6.3 ± 3.5	23.4 ± 5.8	<0.001
Male/female	6/12	10/8	0.18

Abbreviations: DN, diagnostically neutral; DS, diagnostically specific.

**Table 3:** Results of fMRI data analysis: voxel-level *p*  < 0.001 uncorrected, cluster-level pFWE < 0.05.

No.	Brain area	*k*	*T*-value	MNI coordinates		
				*x*	*y*	*z*
Patients > HC (2-sample *t*-test)						
1	R: supramarginal gyrus	119	5.02	36	−46	44
2	R: Precuneus	217	4.70	9	−70	53
3	L: supramarginal gyrus	100	4.47	−36	−49	38
Patients > HC (2-sample *t*-test)—age as covariate						
1	R: Precuneus	123	4.28	6	−70	53

## Data Availability

The data that support the findings of this study are available upon request from the corresponding author. The data are not publicly available due to privacy or ethical restrictions.
